# Public hospital pharmacists’ knowledge, attitudes, and practices for antibiotic stewardship implementation in Limpopo Province, South Africa

**DOI:** 10.1186/s40780-024-00331-3

**Published:** 2024-02-01

**Authors:** Tiyani Comfort Mthombeni, Johanita Riétte Burger, Martha Susanna Lubbe, Marlene Julyan

**Affiliations:** Medicine Usage in South Africa (MUSA), Faculty of Health Sciences, North-West University, Potchefstroom Campus, Potchefstroom, South Africa

**Keywords:** Antibiotic stewardship program, Knowledge, Attitudes, Practices (KAP), Hospital pharmacists, Limpopo Province, South Africa

## Abstract

**Background:**

Hospital pharmacists are important in antibiotic stewardship programs (ASP), a global strategy to combat antibiotic resistance (ABR). South African public hospitals have limited ASP implementation. This study describes Limpopo Provincial Hospital pharmacists’ knowledge, attitudes, and practice toward ASP implementation.

**Method:**

A questionnaire to explore pharmacists’ knowledge, attitudes and practices regarding ASP implementation comprised 43 questions hosted online. A link was sent by invitation e-mail to eligible respondents in November 2021. Five questions on respondents’ demographics, 15 questions on ASP knowledge, 10 Likert scale questions on attitude, and 13 on ASP practices were included. Mean (standard deviation (SD)) knowledge and attitude scores and a median (interquartile range (IQR)) practice score was calculated. Associations between categorical variables were assessed using chi-square/Fisher’s exact analysis (*p* < 0.05), with Cramér’s V as effect size.

**Results:**

The survey yielded 35 responses (13.1%). Twenty (57.1%) respondents were female. Seventeen (48.6%) respondents were between the ages of 31 and 40 years. The mean knowledge score of respondents was 9.8 (2.6) (*N* = 34), with knowledge gaps on *One Health* and socioeconomic determinates of ABR. Ten (29.6%) respondents thought *One Health* discouraged multi-sector collaboration, and nineteen (55.9%) respondents thought ASP was the only strategic response to ABR. Sixteen (47.1%) respondents did not know that poor access to clean water accelerates ABR and seventeen (50.0%) did not know that poverty could be a determinant for antibiotic use. The mean respondent attitude score was 8.0 (1.7) (*N* = 28). Twenty-seven (96.4%) respondents agreed that ASP was necessary and agreed to participate in ASP respectively. All 28 (100.0%) respondents agreed to lead an ASP. The median (IQR) respondents’ practice score was − 2.0 (IQR: -6.0–5.8) (*N* = 16). Respondents were inconsistently (never, sometimes, every time) participating in multi-disciplinary forums (6/16, 37.5%) and expressed a desire for training (11/13, 84.6%) on ASP through in-service (7/27, 25.9%). Respondents thought ASP training should include medical officers (12/29, 41.4%) and nurses (9/29, 31.8%). Knowledge score was associated with gender (*p* = 0.048; *V* = 0.416) and attitude score (*p* = 0.013; *V* = 0.556).

**Conclusion:**

Our study found pharmacists had good knowledge and a positive attitude toward ASP implementation but poor ASP practices. A multi-disciplinary in-service training could address identified knowledge and practice gaps.

**Supplementary Information:**

The online version contains supplementary material available at 10.1186/s40780-024-00331-3.

## Background

Antibiotic stewardship programs (ASP) are an important strategic response to the global public health threat of antibiotic resistance (ABR) [1]. In 2019, ABR was associated with 4.95 million global deaths [2]. Furthermore, ABR results in prolonged or recurrent hospital stays, high healthcare costs, and increased mortality, emphasizing the significance of ASP implementation [1]. Hospital ASPs are multi-disciplinary structures that comprise healthcare leadership, pharmacists, physicians, microbiology and information technology [3], responsible for coordinating activities to improve, measure and encourage access to appropriate antibiotics to reduce ABR [4]. Pharmacists play an essential role in implementing ASPs worldwide, and their contributions have benefited healthcare systems [5–7]. Evidence from low- and middle-income countries in Africa further demonstrates the contributions of pharmacists to ASPs [8; 9], even though early evidence indicated that there was limited ASP implementation in resource-limited settings [10].

Pharmacists are well positioned to act as essential members of hospital ASPs, ensuring that regulatory requirements of ASPs are fulfilled due to their everyday multi-disciplinary collaborations [11]. Pharmacists have an important role in ASPs, transcending the patient care continuum and the healthcare system [5, 6]. This role includes the following: optimizing prescribing behavior in inpatient, ambulatory, and emergency care units, monitoring and evaluating antibiotic use, and the development and review of policies and protocols for antibiotics, infection prevention and control, and integrated rapid diagnostic testing [5, 6, 11, 12]. Moreover, pharmacists also conduct ASP education, training, public engagement, and antibiotic stewardship leadership and coordination in their working environment [5, 6, 11, 12].

Globally, there is inadequate pre-service and in-service education to prepare healthcare professionals for ASP, broad perceptions and attitudes, as well as practice diversity on ASP, leading to inappropriate antibiotic prescribing and use [13, 14]. Comprehensive knowledge and understanding of ASPs are important in implementing ASP interventions [13]. Clinical pharmacists in the public and private sectors of South Africa believe that ASP knowledge is least likely to be acquired during undergraduate pharmacy training because undergraduate programs inadequately prepare pharmacists for their ASP role [14]. Knowledge, attitude, and practices (KAP) studies establish baseline knowledge, myths, misconceptions, attitudes, beliefs, and behaviors related to a specific topic and provide contextualized intervention [15].

Few studies on KAP for ASP implementation have previously been conducted in South Africa [14, 16–18]. These studies have included other health professionals (e.g., medical doctors and nurses), all sectors of pharmacy practice and pharmacy students [14, 16–18], but none have exclusively focused on hospital pharmacists in the public sector. Hospital pharmacists’ perspectives on ASP participation are important, considering the inadequate implementation of the South African national antimicrobial stewardship framework in public sector hospitals [16]. Our study expands on the existing research in the field by assessing hospital pharmacists’ knowledge of socioeconomic determinants of ABR, the novel *One Health* concept, and core elements of hospital ASP. Furthermore, our study aims to assess hospital pharmacists’ attitudes and practices, particularly their role in hospital ASP implementation and their participation in multi-disciplinary clinical governance structures, which are essential for hospital ASP implementation.

## Methods

### Study design and setting

Our study employed a descriptive, cross-sectional design. A self-administered online questionnaire was used to collect data. The South African healthcare system consists of two sectors: the state-financed public sector (~ 80.0%) and the private sector (~ 20.0%) [19]. The private sector is funded by medical schemes or individual out-of-pocket payments [19]. There are significant variations in population medical coverage among provinces, with Western Cape and Gauteng, the two wealthiest provinces, having coverages of 25.1% and 23.9%, respectively [19]. Limpopo Province, the poorest province, had 8.2% of its 6.6 million population covered by medical aid, heavily relying (86.1%) on the public sector for health services [19]. The South African public health system comprises primary health clinics, community health centers and hospitals [20]. The hospitals in the public sector are classified as district, regional, tertiary, central, and specialized (Additional file 1: Supplementary Table 1: Categorization of public sector hospitals in South Africa) [20].

The study respondents were all employed at the 41 public sector hospitals managed by the Limpopo Department of Health [21]. The 41 hospitals are divided according to the South African National Health Act 61 of 2003 regulations into four categories based on their level of care [20]: two tertiary hospitals, five regional hospitals, 30 district hospitals and four specialized hospitals (three mental healthcare long-term hospitals and one tropical diseases hospital) [21].

### Inclusion and exclusion criteria

Total population (*N* = 268) purposive sampling was used, and all pharmacists working in public sector hospitals in Limpopo Province who agreed to participate (*n* = 35) in the study were included. All pharmacists employed in the Limpopo Province pharmaceutical depot (*n* = 12), provincial (*n* = 7) and district offices *(n* = 5), and primary healthcare facilities *(n* = 12) were excluded.

### Questionnaire development

The online questionnaire was developed after reviewing the literature [13, 22–34]. The questionnaire consisted of five questions on respondents’ demographics [22–25] (section A), 15 questions on ASP knowledge [13, 26–27] (section B), 10 Likert scale questions on respondents’ attitudes [28–33] (section C), and 13 questions on ASP practices [23, 25, 26, 34] (section D). The questionnaire combined close-ended (multi-response latent format) and open-ended statements, with space for respondents to contribute additional responses. The questionnaire was reviewed for content, construct and face validity. Additional features reviewed include questionnaire ordering, readability, clarity and comprehensiveness.

### Data collection procedure

Ethical approval of the study was obtained from the North-West University Health Research Ethics Committee (NWU-HREC) (NWU-00312-20-A1). Approval was further obtained from the Head of Department of the Limpopo Department of Health (in accordance with Section 20 subsection (a) of the Protection of Personal Information Act (Act 4 of 2013) [35].

An e-mail list of all public sector pharmacists in Limpopo Province in the most recent (2021) employment record was obtained from the Limpopo Department of Health Human Resource Directorate. The Limpopo Department of Health pharmaceutical services directorate assisted in identifying the practice location of pharmacists (hospital, primary healthcare, provincial and district pharmaceutical offices and pharmaceutical depot).

An independent person e-mailed the study information advertisement and a link to the informed consent form, which included a link to the questionnaire for all eligible respondents. The online questionnaire used the services of the SurveyMonkey® software program.

### Data analysis and assessment of knowledge, attitude, and practice

Data were analyzed using Statistical Package for the Social Sciences (SPSS®) version 28. Not all respondents answered and rated each question from sections B to D. As a result, *N-*values differ for each variable. Limited inferential statistics were performed due to the low respondent response rate. Respondents’ demographic analyses and practices were reported using frequencies. The 15 knowledge statements (“true”, “false”, and “not sure” reply options) were used to determine ASP-related knowledge scores. A correct answer received one point, while the wrong answer or “not sure” received zero. The overall knowledge score was calculated by totaling the scores, with a maximum of 15 per respondent. A total score of 0–7 was considered poor knowledge, 8–12 good, and 13–15 excellent. The mean (standard deviation (SD)) knowledge score was calculated for the respondents. Attitude assessment of the 10 Likert-scale statements was recorded as frequency and proportion of respondents who chose each response continuum choice (agree (strongly agree, agree); neutral; disagree (disagree, strongly disagree)). For the attitude score, one point was given for the eight positively framed questions (agreement (strongly agree, agree) and two negatively framed questions (disagreement (strongly disagree, disagree)) that were considered as a positive attitude. A zero score was given for disagreement (strongly disagree or disagree) with each of the positively framed questions, for agreement (strongly agree or agree) with the two negatively framed questions or “neutral” response, which was considered as a negative attitude. The overall attitude score was calculated by totaling the scores, with a maximum of 10 per respondent. A mean (SD)) attitude score was calculated for the respondents. A score of ≥ 5 was considered positive, and < 5 a negative attitude. The total practice score was determined by aggregating respondents’ responses to seven questions. Participants received two points for “almost every time,” one for “frequently,” zero for “sometimes,” minus one for “almost never,” and minus two for “never,” with a maximum score of ± 14. A total score of ≥ 1 was rated as good and < 1 as poor practice. The median (interquartile range (IQR)) practice score was calculated for the respondents.

The association between respondents’ knowledge, attitude, and practice scores and demographic variables was determined using the chi-square/Fisher’s exact test with a *p*-value of 0.05 for statistical significance. For practical significance, Cramér’s V ≤ 0.2 deemed weak association, 0.2–0.6 deemed moderate association and > 0.6 deemed strong association.

## Results

### Respondents’ demographics

The online questionnaire was distributed to 268 e-mail addresses; 35 respondents responded, yielding a response rate of 13.1%. Table 1 shows that 17 (48.6%) of the respondents were between 31 and 40 years old, 20 (57.1%) were female, and 18 (51.4%) had more than seven years of experience as pharmacists. In terms of practice setting, 17 (51.4%) worked in a district hospital and nine (25.7%) at a tertiary hospital. Twenty-one (60.0%) respondents had a Bachelor of Pharmacy degree, seven (20.0%) had a Master of Pharmacy degree, six (17.1%) had a Bachelor of Pharmacy and a Postgraduate diploma, and one (2.9%) had a Bachelor of Pharmacy and a Master of Public Health degree.

**Table 1:** Respondents’ demographic profile

Demographic variable	Category (*N* = 35)	n	%
Age	21 to 30	10	28.6
	31 to 40	17	48.6
	41 to 50	5	14.3
	41 to 60	3	8.6
Gender	Male	15	42.9
	Female	20	57.1
Number of years practicing as a pharmacist	Entry level into practice (0 to 3)	4	11.4
	Intermediate practice (4 to 7)	13	37.1
	Advanced practice (more than 7)	18	51.4
Category of hospital currently practicing under	District	17	48.6
	Regional	7	20.0
	Specialized	2	5.7
	Tertiary	9	25.7
Highest level of academic qualification attained	Bachelor of Pharmacy and Postgraduate Diploma	6	17.1
	Bachelor of Pharmacy	21	60.0
	Master of Pharmacy	7	20.0
	Other (Master of Public Health)	1	2.9

*N*: population; *n*: frequency

### Knowledge of antibiotic resistance, use and stewardship programs

The mean (SD) knowledge score of respondents was 9.8 (2.6), with 28 (82.4%) having good knowledge of antibiotic resistance, use, and stewardship. The respondents’ knowledge score was independent of age (*p* = 0.382), number of years practicing as a pharmacist (*p* = 0.438), category of hospital currently practicing under (*p* = 0.741), highest level of academic qualification attained (*p* = 0.092) and practice score (*p* = 0.233). Knowledge score was moderately associated with gender (*p* = 0.048; Cramér’s V = 0.416) and attitude score (*p* = 0.013; Cramér’s V = 0.556). Table 2 presents data on the questions to assess respondents’ knowledge of antibiotic resistance, use, and stewardship (*N* = 34). Thirty-three (97.1%) respondents were aware that antibiotics are ineffective against viruses, 23 (67.6%) were aware that ABR is a natural occurrence, and 29 (85.3%) were aware that excessive antibiotic use accelerates the development of resistance. Sixteen (47.1%) respondents were unaware that poor access to clean water accelerates ABR development, and 17 (50.0%) did not know that poverty could be a determinant for antibiotic use. Ten respondents (29.4%) indicated that the *One Health* concept discourages multi-sector collaboration to limit ABR, and 19 (55.9%) indicated that ASP was the only strategy designated to address ABR.

**Table 2:** Respondents’ knowledge of antibiotic resistance, use and stewardship

Knowledge statement (*N* = 34)	Correct answer	True		Not sure		False	
	True or False	*n*	%	*n*	%	*n*	%
Antibiotics are effective against viruses	False	1	2.9	0	0.0	33	97.1
Antibiotic resistance is a natural event	True	9	26.5	2	5.9	23	67.6
Excessive use of antibiotics in humans hastens the development of antibiotic resistance	True	29	85.3	3	8.8	2	5.9
Poor access to clean water accelerates the development of antibiotic resistance	True	12	35.3	6	17.6	16	47.1
The *One Health* concept discourages multi-sectorial collaboration to limit antibiotic resistance	False	10	29.4	11	32.4	13	38.2
Antibiotic stewardship program is the only strategy designated to address antibiotic resistance	False	19	55.9	5	14.7	10	29.4
One of the primary objectives of an antibiotic stewardship program is to improve patient safety	True	33	97.1		0.0	1	2.9
Reporting on antibiotic use is one of the core components of the antibiotic stewardship program	True	30	88,2	3	8.8	1	2.9
Underuse is a type of antibiotic misuse	True	25	73.5	2	5.9	7	20.6
Organizational cultures, attitudes, beliefs and poor knowledge are some of the key determinants of antibiotic use	True	31	91.2		0.0	3	8.8
Physicians, pharmacy staff and nurses are the only stakeholders in an antibiotic stewardship program	False	3	8.8	3	8.8	28	82.4
Pharmacokinetics are irrelevant in an antibiotic stewardship program	False	0	0.0	1	2.9	33	97.1
Monitoring expenditure cost of antibiotic use cannot be used to assess the effectiveness of an antibiotic stewardship program	False	4	11.8	6	17.6	24	70.6
Politicians and the media can play a critical role in antibiotic stewardship program communication	True	25	73.5	4	11.8	5	14.7
Poverty is one of the key determinants of antibiotic use	True	9	26.5	8	23.5	17	50.0

*N*: population; *n*: frequency

### Attitude to antibiotic stewardship programs

The respondents’ mean attitude score was 8.0 (1.7). The attitude score was independent of the respondents’ age (*p* = 0.240), gender (*p* = 0.309), number of years practicing as a pharmacist (*p* = 0.638), category of hospital currently practicing under (*p* = 0.284), highest level of academic qualification attained (*p* = 0.284) and practice score (*p* = 0.424). Table 3 presents respondents’ agreement with ASP statements to assess their attitude (*N* = 28). Twenty-two (78.6%) respondents agreed that ABR was a problem in their hospital, whereas 27 (96.4%) respondents believed that ASP was key to reducing ABR. Most respondents agreed pharmacists should participate in ASP, implement successful ASP (27/28, 96.4%), and lead ASP policy development (28/28, 100.0%). Eighteen respondents (64.3%) disagreed that only a trained clinical pharmacist can implement a functional ASP, and 15 (53.6%) disagreed that ASP can only be implemented in well-resourced institutions. Fifteen (53.6%) other respondents agreed that sourcing rapid diagnostic tests is a pharmacist’s role. Twenty-five (89.3%) respondents agreed that nurses play an important role in ASP.

**Table 3:** Respondents’ attitudes on antibiotic resistance, use and stewardship program

Attitude statement (*N* = 28)	Strongly Agree		Agree		Neutral		Disagree		Strongly disagree	
	*n*	%	*n*	%	*n*	%	*n*	%	*n*	%
Antibiotic resistance is a problem in your hospital	12	42.9	10	35.7	3	10.7	2	7.1	1	3.6
Empirical antibiotic prescribing accelerates the initiation of patient treatment	9	32.1	11	39.3	7	25.0	1	3.6	0	0.0
Antibiotic stewardship activities are key to the reduction of antibiotic resistance	24	85.7	3	10.7	1	3.6	0	0.0	0	0.0
Pharmacists must participate in antibiotic stewardship program activities	23	82.1	4	14.3	0	0.0	1	3.6	0	0.0
Pharmacists can implement a successful antibiotic stewardship program intervention	23	82.1	3	10.7	2	7.1	0	0.0	0	0.0
Pharmacists can lead to antibiotic stewardship program policy development	22	78.6	6	21.4	0	0.0	0	0.0	0	0.0
Sourcing of rapid diagnostic tests is one of the key roles of a pharmacist in an antimicrobial stewardship program	9	32.1	6	21.4	5	17.9	7	25.0	1	3.6
Only a trained clinical pharmacist must lead an antibiotic stewardship program	2	7.1	6	21.4	2	7.1	13	46.4	5	17.9
A functional antibiotic stewardship program is only possible in a well-resourced institution	2	7.1	11	39.3	0	0.0	9	32.1	6	21.4
Nurses play a critical role in antibiotic stewardship program activities	17	60.7	8	28.5	1	3.6	1	3.6	1	3.6

*N*: population; *n*: frequency

### Antibiotic stewardship program practices

The median practice score for the respondents was − 2.0 (IQR: -6.0–5.8) (*N* = 16). The practice score was independent of the respondents’ age (*p* = 0.074), gender (*p* = 0.424) number of years practicing as a pharmacist (*p* = 0.701), category of hospital currently practicing under (*p* = 0.917) and highest level of academic qualification attained (*p* = 0.488).

The respondents were asked to self-report their participation frequency in multi-disciplinary clinical forums (never or almost never or sometimes or frequently or almost every time). Seven of the 16 respondents worked in district hospitals, three in regional hospitals, two in specialized hospitals, and four in tertiary hospitals. Six of 16 respondents have never attended the multi-disciplinary clinical ward rounds, ASP committee, or patient safety meetings, whereas another six have attended the pharmacy and therapeutic committee meetings almost every time (Table 4). Six respondents sometimes attended the Infection Prevention and Control Committee meetings and the Morbidity and Mortality Committee.

**Table 4:** Respondents’ multi-disciplinary forum participation

Variable (*N* = 16)	Never		Almost never		Sometimes		Frequently		Almost every time	
	*n*	%	*n*	%	*n*	%	*n*	%	*n*	%
Pharmacy and Therapeutics Committee meetings	2	12.5	0	0.0	4	25.0	4	25.0	6	37.5
Multi-disciplinary clinical ward-rounds	6	37.5	3	18.8	4	25.0	1	6.3	2	12.5
Antimicrobial stewardship committee	6	37.5	3	18.8	4	25.0	2	12.5	1	6.3
Infection prevention and control committee	4	25.0	1	6.3	6	37.5	2	12.5	3	18.8
Patient safety committee	6	37.5	2	12.5	3	18.8	2	12.5	3	18.8
Morbidity and mortality meetings	4	25.0	2	12.5	6	37.5	2	12.5	2	12.5
Local continued professional development sessions	3	18.8	2	12.5	5	31.3	3	18.8	3	18.8

*N*: population; *n*: frequency

The study respondents were requested to mark multiple options for the top three ASP interventions when implementing ASP. Adherence to treatment guidelines and clinical pathways (12/45, 26.7%), dose optimization (10/45, 22.2%), and parenteral to oral conversion (7/45, 15.6%) were the top three preferred ASP interventions (Table 5). Respondents in the study were required to describe their ASP-related training requirements, training format, and the involvement of other healthcare professionals in ASP training (Table 5). Most respondents (11/13, 84.6%) desired additional training. Such training could be offered through in-service (7/27, 25.9%) and workshop formats (6/27, 22.2%). Respondents preferred that doctors (12/29, 41.4%) and nurses (9/29, 31.0%) participate in ASP education.

**Table 5:** Respondents’ various antibiotic stewardship program implementation practices

Variable	n	%
Top three interventions (*N* = 45)*		
Adherence to guidelines and clinical pathways	12	26.7
Dose optimization	10	22.2
Parenteral to oral conversion	7	15.6
Antimicrobial order forms	6	13.3
Education	6	13.3
Streamline or de-escalation therapy	4	8.9
Closed formulary	0	0.0
**Communication of prescribing and antimicrobial use feedback (*N*** = 28)*		
Staff meetings	8	28.6
Clinical meetings (e.g., morbidity and mortality)	8	28.6
Special feedback session	6	21.4
Booklet	4	14.3
Other (Please specify) 1: WhatsApp group	1	3.6
Other (Please specify) 2: written report	1	3.6
Newsletter	0	0.0
**Frequency of feedback sessions on antibiotic prescribing and use (*N*** **= 15)**		
Monthly	10	66.7
Quarterly	5	33.3
Twice a year	0	0.0
Once a year	0	0.0
**Measuring the effectiveness of antibiotic stewardship programs (*N*** = 22)*		
Antimicrobial resistance	9	40.9
Antimicrobial expenditure	8	36.4
Frequency of medical officers’ acceptance of AMS recommendations	5	22.7
**Format for remote specialized support to remote resource-limited hospitals (*N*** = 20)*		
Regular personal support visits by infectious disease physician	10	50.0
Off-line smart-phone applications (Apps)	4	20.0
Telephone remote support	3	15.0
Tele-conferencing	3	15.0
**Additional training need*(N*** **= 13)**		
Yes	11	84.6
No	1	7.7
Not sure	1	7.7
**Learning areas (*N*** = 32)*		
General antimicrobial principles	8	25.0
Microbiological and laboratory data	7	21.9
Pharmacokinetics and pharmacodynamics effects on antimicrobial stewardship	7	21.9
Role of a pharmacist in antimicrobial stewardship	6	18.8
Approaches used in antimicrobial stewardship	4	12.5
**Format of additional training do you prefer (*N*** = 27)*		
In-service training	7	25.9
Workshop	6	22.2
Off-site short course	5	18.5
Postgraduate diploma	5	18.5
Postgraduate certificate	4	14.8
**Training sessions on antibiotic stewardship be assessed (*N*** = 21)*		
Pre- and post-training test	9	42.9
Case scenarios	7	33.3
Role-play simulation	3	14.3
Post-training quiz	2	9.5
**Mode of training on antibiotic stewardship (*N*** = 19)*		
Face-to-face	7	36.8
Short courses	7	36.8
E-learning	4	21.1
Written information provided	1	5.3
**Source of information on antibiotic stewardship over the last 12 months (*N*** **= 14)**		
Internet website	5	35.7
Smart-phone application (App)	4	28.6
None of the above	4	28.6
Other: National Department of Health guidelines	1	7.1
University Library	0	0.0
Social media	0	0.0
**Hospital healthcare workers to be involved in antibiotic stewardship education (*N*** = 29)*		
Medical officers	12	41.4
Nurses	9	31.0
Laboratory technicians	5	17.2
Administrators	1	3.4
Information and records personnel	1	3.4
Other (Please specify): Dentists	1	3.4

* Multiple answers were possible; N: population; *n*: frequency

## Discussion

The study aimed to assess Limpopo Province public hospital pharmacists’ KAP in ASP implementation using an online questionnaire. We found that the respondents from the public hospitals (pharmacists) had good knowledge of antibiotic resistance, use, and stewardship, with gaps in *One Health* and socioeconomic determinants of ABR. Respondents were willing to participate in and lead ASPs and had positive attitudes toward ASPs. Respondents were inconsistently participating in multi-disciplinary clinical forums but were keen on multi-disciplinary ASP training through in-service. Respondents had good knowledge and positive attitudes toward, but poor ASP practices.

This study confirmed findings from other South African studies that found most respondents had good knowledge and correctly answered knowledge statements about antibiotic resistance, use, and stewardship, such as antibiotics are not effective in treating viral infections (92.6%) [17] and most (71.8%) respondents were familiar with ASP [18]. Balliram et al. [17]. and Burger et al. [18]. ascribe this observed good knowledge of antibiotic resistance, use and stewardship program to most respondents being aware that antibiotics are ineffective against viruses, that ABR is a natural process, that excessive antibiotic usage contributes to ABR, that the main purpose of an ASP is to improve patient safety, that reporting antibiotic usage is important, that pharmacokinetics are crucial, and that monitoring antibiotic expenditure cost is an ASP activity.

The observed knowledge gaps included respondents’ lack of understanding of the *One Health* concept, the existing relationship between lack of clean water, poverty, and the development and spread of ABR, as well as various strategies (e.g., infection prevention and control, water, sanitation and hygiene, vaccination) to respond to ABR along ASP [1]. *One Health* is a collaborative, multisectoral and transdisciplinary approach that recognizes the interconnection between humans, animals, plants and their shared environment [1, 3, 36, 37]. Globally, ABR affects humans, animals, and the environment [37]. Antibiotic misuse in agriculture, animals, and human health impacts all three *One Health* components and the migration of humans and animals with resistant infection promotes resistance [37–39]. Global ecological country-level data suggest that socioeconomic determinants of health, such as governance, poverty, and access to water and sanitation, are significantly linked and associated with the development of ABR in humans, animals, and food [38, 40]. Therefore, ABR prevention and control methods should prioritize poverty reduction and aim to prevent ABR transmission across various *One Health* domains while considering domain-specific risk factors such as healthcare system quality, water sanitation and hygiene, gross domestic product per capita, and climate [40, 41]. Environmental components (air, soil and water) in *One Health* serve as reservoirs and transmission pathways of ABR [41].

In terms of attitude, most respondents had a positive attitude towards ASP. Most (> 70.0%) respondents agreed that ABR was a problem in their hospitals, that ASP was essential in reducing ABR and that pharmacists should participate in ASP and lead ASP policy development. Pharmacists are custodians of medicines and can lead the ASP interventions through audits and building relationships with prescribers to influence decisions about prescribing [42]. There is sufficient global [5, 7], regional [8], and national [43, 44] (including the public sector) evidence to confirm that pharmacists have implemented and led ASPs. Over two-thirds of respondents disagreed that only a trained clinical pharmacist can implement a successful ASP, and over half disagreed that ASP can only be implemented in well-resourced institutions. There is evidence corroborated by respondents’ suggestions in South African public sector hospitals, where non-clinical pharmacists have participated, collaborated, and led ASP interventions with positive outcomes for patients in resource-constrained settings [43, 44].

Most respondents (89.2%) agreed that nurses play an important role in ASP. Evidence suggests that formal involvement of nurses in ASP initiatives improves nurses’ knowledge, confidence, partnership with other professionals and patient outcomes [45, 46]; therefore, hospital pharmacists’ embracing nurses’ roles is essential and could encourage a multi-disciplinary team approach in ASP implementation, particularly in low-resourced, small, and rural hospitals of Limpopo Province [47]. Over half of the respondents in this study agreed that sourcing rapid diagnostic tests is a pharmacist’s role in ASP. This is an example of diagnostic stewardship. Diagnostic stewardship seeks to reduce the use of unnecessary tests to prevent antibiotic overuse [48].

According to the International Pharmaceutical Federation, hospital pharmacists should be integral members of multi-disciplinary teams responsible for therapeutic decision-making in all patient care domains [49]. In this study, pharmacists showed poor and inconsistent practice as an equal number of respondents (< 40.0%) have never attended an ASP committee meeting, have sometimes attended infection prevention and control committee meetings, and have almost always attended pharmacy and therapeutic committee meetings, indicating inconsistent attendance among clinical governance structures. Similar to prior research conducted in South Africa, pharmacists in Limpopo Hospitals expressed a desire for training in ASP [14, 16–18]. The inadequate coverage of the ASP subject in South Africa’s undergraduate pharmacy education curricula may account for the high demand for ASP training among study respondents [14, 18]. A recent South African study on clinical pharmacists found that ASP education was inadequate in undergraduate pharmacy programs and required additional training through master’s programs, short courses, continued professional development, and workshops [14]. Moreover, education and training are key components of the hospital ASP implementation framework [1, 13].

Finally, in this study, hospital pharmacists ranked guideline adherence as a key ASP intervention they would prioritize in ASP implementation, which may necessitate education-based interventions (proven to improve guideline adherence) aimed at prescribers [7]. Education-based interventions are preferable on ASP inception since they are resource-efficient and appropriate in limited resource settings [1, 7].

In contrast to our findings, a nationwide Australian study found that more years of working as a pharmacist and post-graduate qualification were associated with good antimicrobial stewardship knowledge [50]. This comparison should be interpreted with caution since it may represent differences in antibiotic stewardship education and practice exposure in Australia and South Africa [50]. Contrary to our findings, global evidence suggests that a positive attitude toward antibiotic stewardship is associated with post-graduate education and more years of working as a pharmacist [51]. The moderate association between gender and knowledge in our study could be attributed to response bias since most of our respondents were female (20/34; 57.1%). The moderate correlation between knowledge and attitude in our study could be attributed to voluntary response bias, as only respondents with good knowledge and a positive attitude participated in the study.

The following limitations are noted for our study: First, a KAP questionnaire can be misleading because it does not address the full complexity of an issue [16]. A complementary qualitative study (e.g., focus group discussion) is recommended to understand KAP and tailor interventions to local realities. Second, the KAP questionnaire study design is prone to social desirability, demand characteristics (e.g., study title or tools) that can alert respondents to the study objectives, and extreme responses (respondents’ tendency to choose only the highest or lowest response). This study attempted to mitigate these biases by adding an option for “other (please specify)”, allowing a fill-in open-ended text and organizing questions by topic and using a combination of positive and negative knowledge and attitude statements. Third, even though our study used personalized email invitations and regular reminders to increase the response rate, the study had a low response rate (13.1%); therefore, its findings could not be representative and generalized to the study population. It is also possible that voluntary response bias may have occurred in the study in that only those with a high knowledge level and attitude participated, as observed in our study. Finally, our study experienced attrition bias, which occurs when respondents get tired and do not complete the questionnaire.

## Conclusions

Our study found that the pharmacists who participated in the study had good knowledge of antibiotic resistance, use, and stewardship. However, they had limited knowledge in some areas, including the novel *One Health* concept to combat ABR and the socioeconomic determinants of ABR. The pharmacists had positive attitudes as they were willing to engage in and lead hospital ASPs, demonstrating their role as core members and essential stakeholders in multi-disciplinary hospital ASPs coordinating optimal antibiotic use. However, they demonstrated poor practices by inconsistently participating in multi-disciplinary clinical governance structures; this lack of collaboration with other structures and stakeholders may become a barrier to hospital ASP implementation. A multi-disciplinary in-service training program has the capacity to address the identified gaps in knowledge and substandard practices.

### Electronic supplementary material

Below is the link to the electronic supplementary material.

**Supplementary Material 1: Supplementary Table 1.** Categorization of public sector hospitals in South Africa, including information on bed capacity, health professional training and research requirements, referral and catchment areas, and medical specialist services

## Data Availability

The datasets used and/or analyzed during the current study are available from the corresponding author upon reasonable request.

## Abbreviations

ABR: Antibiotic resistance
ASP: Antibiotic stewardship program
KAP: Knowledge, attitude and practices
SD: Standard deviation
