# Supplementary material for: Public hospital pharmacists’ knowledge, attitudes, and practices for antibiotic stewardship implementation in Limpopo Province, South Africa
Source: J Pharm Health Care Sci. 2024 Feb 1;10:9. doi: 10.1186/s40780-024-00331-3 (PMC10832237; doi:10.1186/s40780-024-00331-3)
Supplement: Supplementary file 1 — Supplementary Material 1: Supplementary Table 1. Categorization of public sector hospitals in South Africa, including information on bed capacity, health professional training and research requirements, referral and catchment areas, and medical specialist services [file 40780_2024_331_MOESM1_ESM.docx]

Supplementary Table 1: Categorization of public sector hospitals in South Africa

| Hospital type | Definition | Medical specialists’ services | Referral and catchment area | Bed capacity | Training and research |
| --- | --- | --- | --- | --- | --- |
| District | It provides a district hospital package of care on a 24-hour basis using general practitioners and clinical nurse practitioners.  Its services include in-patient, out-patient and emergency health services. | May only provide specialist services  in pediatrics, obstetrics and gynecology, internal medicine, general surgery and family medicine. | It serves a defined population within a health district and supports primary healthcare and receives outreach and support from general specialists based at regional hospitals. | Small: between 50 and 150;  medium: between 150 and 300; and  large district: between 300 and 600. | Where practical, provide training for healthcare professionals. |
| Regional | It provides 24-hour health services in the fields of internal medicine, pediatrics, obstetrics and gynecology, and general surgery; and health services in at least one of the following specialties: orthopedic surgery, psychiatry, anesthetics, diagnostic radiology, trauma and emergency services and short-term ventilation in a critical care unit. | | Receives referrals from various district hospitals and outreach and support from tertiary hospitals for a provincial drainage population. | Between 200 and 800. | Where practical, provide training for healthcare professionals. |
| Tertiary | It provides specialist-level services offered by regional hospitals and their subspecialties, as well as critical care services under the supervision of a specialist or specialist intensivist. | | Receives referrals from regional hospitals not limited to provincial boundaries. | Between 400 and 800. | May provide training for healthcare professionals. |
| National | It provides tertiary hospital services central referral services and may provide national referral services. Central referral services refer to highly specialized units that require unique, skilled and scarce personnel at a small number of sites nationwide. National referral services are super-specialized national referral units for expensive services such as organ transplants (e.g., heart, lung, bone marrow, liver and cochlear implants). | | May receive referrals from all provinces in the country. | Maximum of 1200. | It is attached to a medical school and serves as the primary teaching and research platform for healthcare professionals. |
| Specialized | It provides specialized health services like psychiatric services, tuberculosis services, infectious diseases and rehabilitation services. | | May receive referrals from across the province. | Maximum of 600. | Where practical, provide training for healthcare professionals. |
